# Fabrication of CIS Absorber Layers with Different Thicknesses Using A Non-Vacuum Spray Coating Method

**DOI:** 10.3390/ma7010206

**Published:** 2014-01-03

**Authors:** Chien-Chen Diao, Hsin-Hui Kuo, Wen-Cheng Tzou, Yen-Lin Chen, Cheng-Fu Yang

**Affiliations:** 1 Department of Electronic Engineering, Kao Yuan University, Kaohsiung 82151, Taiwan; E-Mail: t10025@cc.kyu.edu.tw; 2 Department of Electrical Engineering, National University of Kaohsiung, Kaohsiung 81148, Taiwan; E-Mail: hhkuo@nuk.edu.tw; 3 Department of Electro-Optical Engineering, Southern Taiwan University, Tainan 71005, Taiwan; E-Mail: wjtzou@mail.stust.edu.tw; 4 Department of Chemical and Materials Engineering, National University of Kaohsiung, Kaohsiung 81148, Taiwan; E-Mail: cpr4511@hotmail.com

**Keywords:** CuInSe_2_ absorber layer, spray coating method, non-vacuum method, thickness

## Abstract

In this study, a new thin-film deposition process, spray coating method (SPM), was investigated to deposit the high-densified CuInSe_2_ absorber layers. The spray coating method developed in this study was a non-vacuum process, based on dispersed nano-scale CuInSe_2_ precursor and could offer a simple, inexpensive, and alternative formation technology for CuInSe_2_ absorber layers. After spraying on Mo/glass substrates, the CuInSe_2_ thin films were annealed at 550 °C by changing the annealing time from 5 min to 30 min in a selenization furnace, using N_2_ as atmosphere. When the CuInSe_2_ thin films were annealed, without extra Se or H_2_Se gas used as the compensation source during the annealing process. The aim of this project was to investigate the influence of annealing time on the densification and crystallization of the CuInSe_2_ absorber layers to optimize the quality for cost effective solar cell production. The thickness of the CuInSe_2_ absorber layers could be controlled as the volume of used dispersed CuInSe_2_-isopropyl alcohol solution was controlled. In this work, X-ray diffraction patterns, field emission scanning electron microscopy, and Hall parameter measurements were performed in order to verify the quality of the CuInSe_2_ absorber layers obtained by the Spray Coating Method.

## 1. Introduction

CuInSe_2_ (abbreviates as CIS) and Cu(In,Ga)Se_2_ (CIGS) are two of the most promising materials for thin film photovoltaic devices as they have an appropriate direct band gap and high absorption coefficient in the visible and near-infrared lights, and, therewith, low layer thickness required for light absorption. The resultant potential for cost reduction, light-weight, and flexible applications makes CIS and CIGS materials the all-round candidates for cheap, large-area module technology, as well as special architectural and space applications [1]. Many different manufacturing processes have been developed to fabricate the CIS and CIGS absorber layers [2,3], and, thus far, the two-stage method is the cheapest and easiest to perform on an industrial scale [4]. For a two-stage method, the compositional uniformity and surface morphology of metallic precursors affect the quality of the absorber layers. To ensure the compositional uniformity of absorber layers, many procedures using metal or alloy thin films, with differently stacked Cu-In and Cu-In-Ga structures, have been proposed [5,6].

In addition to the two-stage growth processes, sputtering and co-evaporation are two of the most popular vacuum methods to deposit CIS and CIGS absorber layers. Shi *et al.* used one-step sputtering of a single quaternary CIGS chalcogenide target at room temperature to fabricate the CIGS thin films. However, the CIGS thin films were also followed by a post-selenization process to get the CIGS absorber layers using Se vapor, obtained from elemental Se pellets [7]. M. Powalla *et al.* used the co-evaporation process to fabricate highly efficient CIS absorber layers and solar cells [1]. P. Guha *et al.* also used the co-evaporation process to fabricate highly efficient CIGS absorber layers on different substrates [8]. When the number of process parameters increases, the traditional two-stage method and vacuum methods are too complicated and difficult [9,10]. Several vacuum-manufacturing processes have been developed, however, those methods need expensive deposition and vacuum equipment and large amount of CIS or CIGS precursors are lost during the deposition processes. For that, investigating a non-vacuum and simple method to increase experimental efficiency of formed CIS and CIGS absorber layers, and allow the design of products with improved quality and low cost, is an important issue for fabricating the CIS- and CIGS-based solar cells.

In the past, Saji *et al.* used the electrodeposited method to deposit CIGS absorber layers in a non-vacuum atmosphere [9]. In addition, Eberspacher *et al.* used the sub-micron particulate materials to deposit the CIS absorber layers in a non-vacuum atmosphere [10] and then densified into polycrystalline CIS absorber layers by a post-heating process. The spray pyrolysis method (SPM) is a very important non-vacuum deposition method to fabricate thin films as it is relatively simple and it can use an inexpensive non-vacuum deposition method for large-area coating [11]. In this study, the new SPM method will offer a simple, inexpensive, and alternative method to fabricate the CIS absorber layers with high densification structures. We would systematically investigate the effects of different thermal-treated time in a selenization furnace on the physical and electrical properties of the CIS absorber layers. The paper focuses on nano-scale-based particles and a non-vacuum technique to form the CIS absorber layers with different thicknesses for thin film solar cell application in the future.

## 2. Experimental Procedures

**Figure 1.**
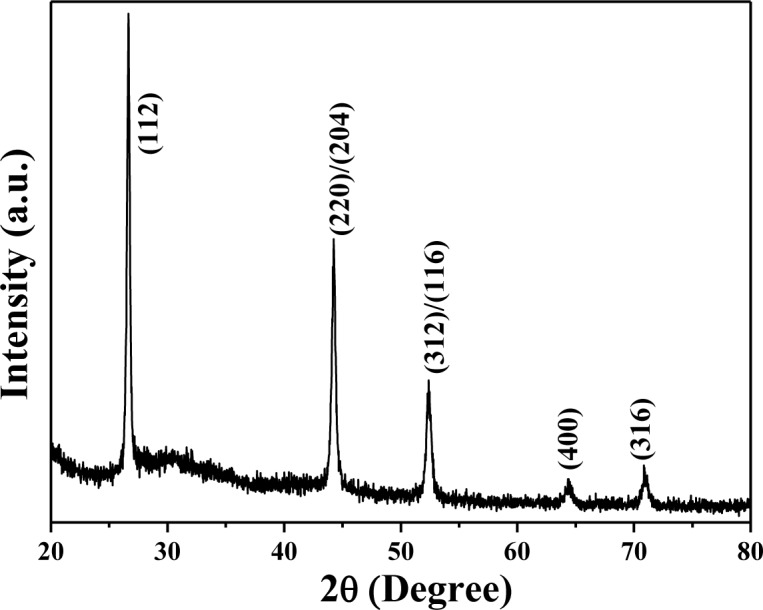
X-ray pattern of the synthesized CIS precursor.

**Figure 2.**
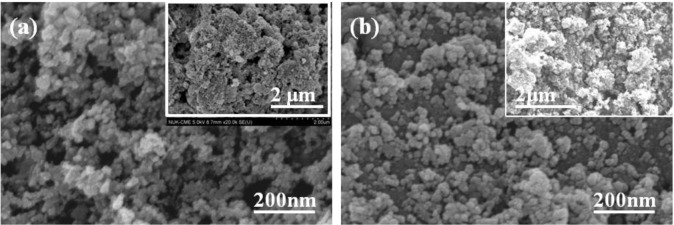
Micrographs of (**a**) the synthesized CIS powder; (**b**) grinding and baking CIS power.

High purity CuInSe_2_ (Cu:In:Se = 25:27:48, CIS) was synthesized using hydrothermal process, by Nanowin Technology Co. Ltd. The CIS precursors were examined by using an X-ray diffraction (XRD) pattern, and Figure 1 shows XRD pattern of the synthesized CIS powder. The diffraction peaks of (112), (220)/(204), (312)/(116), (400), and (316) planes were found in CIS powder. The XRD pattern proves the tetragonal chalcopyrite phase was formed accompanying amorphous phase in the hydrothermally synthesized CIS powder, and no secondary phases were observed. The morphologies of the CIS precursors before and after being dispersed were examined by using the field-emission scanning electron microscope (FE-SEM). The originally synthesized CIS powder was in nano-scale, with particle sizes approximately 40–80 nm (Figure 2a). However, the nano-scale CIS powders were aggregated into micro-meter scale particles, and the particle sizes were approximately 5–15 μm, as the inset shown in Figure 2a. They could not be used as the source materials to form the CIS absorber layers by using the SPM. Even the ground CIS precursor was also aggregated into larger particles after baking (inset in Figure 2b), the CIS powder was easily dispersed into nano-scale particles in isopropyl alcohol (IPA) and its particle sizes were in the range of 15–35 nm, as the inset in Figure 2b shows.

The organic/CIS composite thin films were formed by SCM on Mo/glass substrates, then they were annealed by using rapid temperature annealing (RTA) process in a selenization furnace (the chamber size is 5 cm × 5 cm × 4 cm) at 550 °C to remove the used organic material and to crystallize the CIS absorber layers. The annealing time was changed from 5 min to 30 min without extra Se powder or H_2_Se gas added in the furnace for compensation during the annealing process. The Energy After annealing process, the crystalline structure was examined again by using an XRD pattern and the surface morphology and cross section observations were examined by using FE-SEM, respectively. Dispersive Spectrometers (EDS) were used to analyze the CIS absorber layers, no residual carbon was detected. Finally, the electrical resistivity and the Hall-effect coefficients were measured using a Bio-Rad Hall set-up.

## 3. Results and Discussion

**Figure 3.**
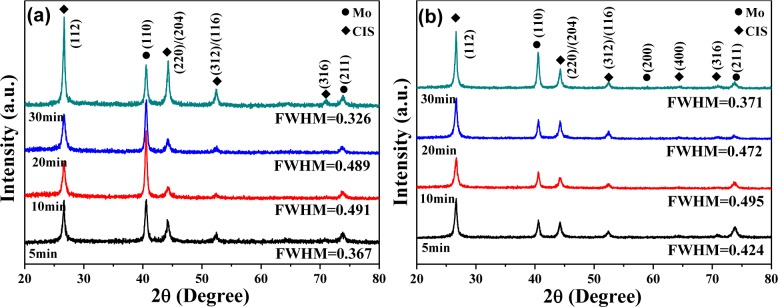
XRD patterns of the CIS absorber layers as a function of annealing time, with using solution volume was (**a**) 0.05 mL; and (**b**) 0.1 mL; respectively.

XRD patterns of the CIS absorber layers on Mo/glass substrates with different volumes of CIS isopropyl alcohol (IPA/CIS) solution and different annealing times are shown in Figure 3. The diffraction peaks of (112), (220)/(204), (312)/(116), (400), and (316) planes were found in all CIS absorber layers. Only the tetragonal chalcopyrite structure was observed, and the amorphous phase was not observed, even though the annealing time was only 5 min. However, the CIS absorber layers exhibited no prominent series of diffraction peaks at other 2θ values, which were not generated by chalcopyrite CIS phase. The absence of additional peaks in the XRD patterns excludes the possibility of any extra phases in the CIS absorber layers. The (112) peak of the all CIS absorber layers was situated at 2θ = 26.66°, independent of annealing time, and the volume of the IPA/CIS composite. In general, the crystallization of CIS absorber layers is highly dependent on the annealing parameters. For that, the crystallization of the CIS absorber layers also strongly depends on the annealing time. Figure 3 also shows that the diffraction intensity of the (112) plane first decreased, as the annealing time was increased from 5 min to 10 min, then it obviously increased with increasing annealing time and reached a maximum at 30 min. As 0.05 mL (0.1 mL) IPA/CIS composite was used and annealing time was 5 min, 10 min, 20 min, and 30 min, the full width at half maximum (FWHM) value for the (112) peak of the CIS absorber layers was 0.367 (0.424), 0.491 (0.495), 0.489 (0.472), and 0.326 (0.371), respectively.

**Figure 4.**
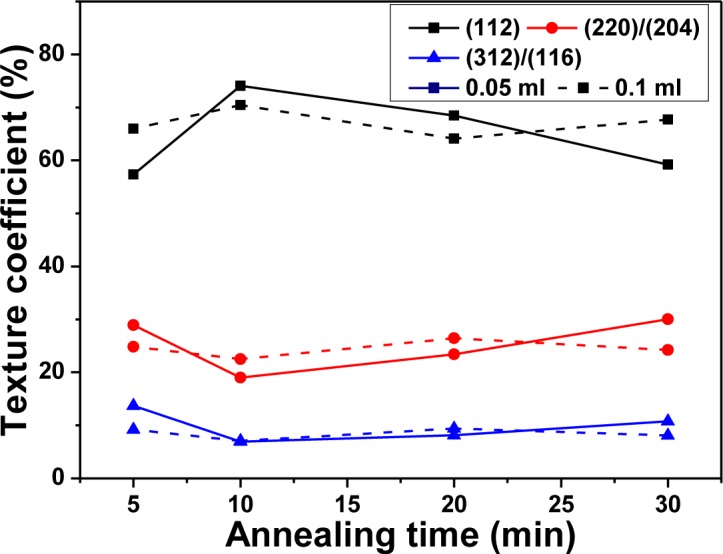
Texture coefficients of the CIS absorber layers a function of annealing time and using solution volume.

Figure 3 shows that all the CIS absorber layers had the polycrystalline structure and included the (112), (220)/(204), (312)/(116), (400), and (316) diffraction peaks, which indicate the crystallographic planes in the chalcopyrite structure. To material scientists, the term texture means the distribution of crystallographic orientations of a polycrystalline sample. The degree of crystallographic texture, which is believed to have a great influence on material properties, is dependent on the percentage of crystals having the preferred orientation. Crystallographic texture of the CIS absorber layers is referred, and texture coefficient (TC) is used to describe the textures of thin films with Equation (1) [12]:

$${\text{ TC}}_{\left(hkl\right)}=\frac{I_{\left(hkl\right)}}{\sum^{\text{​}}I_{\left(hkl\right)}}\times 100\%$$ (1)

where *h*, *k*, and *l* are the Miller indices; TC_(_*_hkl_*_)_ is the TC value of specific (*hkl*) plane; I_(_*_hkl_*_)_ is the measured peak intensity; and Σ*I*_(_*_hkl_*_)_ is the summation of the intensities for the (112), (220)/(204), and (312)/(116) peaks of the CIS absorber layers. The TC values of various reflections of the CIS absorber layers shown in Figure 4 indicate that although the TC value of (112) plane changed with increasing annealing time, the (112) plane possessed the highest TC value at the annealing time of 5 min–30 min, independent of the volume of IPA/CIS composite. As 0.05 mL IPA/CIS composite was used, the TC value of (112) plane first increased, reached a maximum at 10 min, then decreased with further increasing in annealing time. Conversely, as 0.1 mL IPA/CIS composite was used, the TC value of (112) plane had no apparent change.

**Figure 5.**
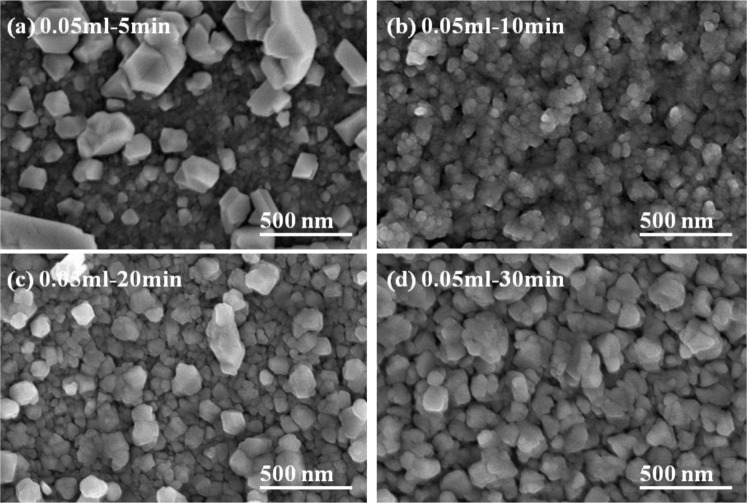
Surface morphologies of the CIS absorber layers as a function of annealing time, the using solution volume was 0.05 mL. (**a**) 5 min; (**b**) 10 min; (**c**) 20 min; (**d**) 30 min.

**Figure 6.**
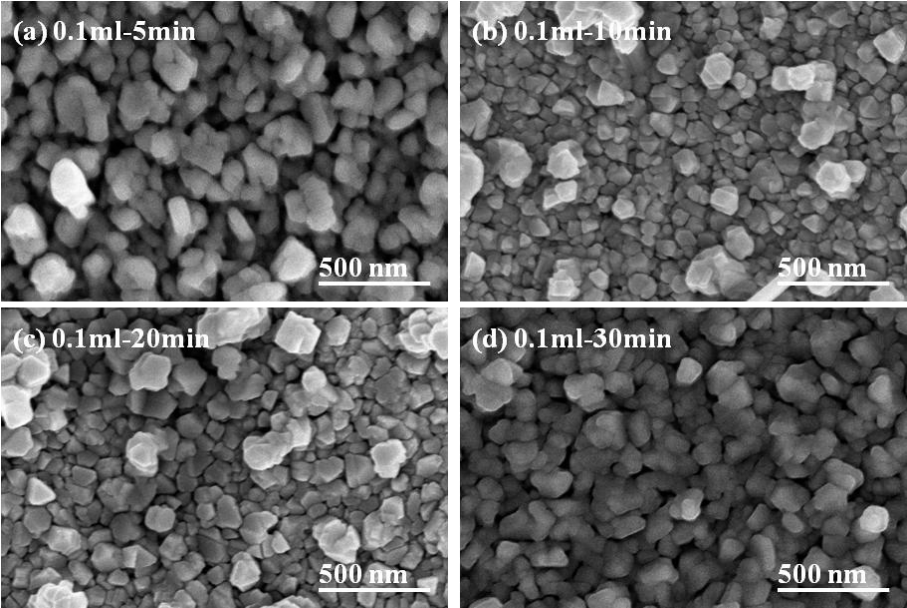
Surface morphologies of the CIS absorber layers as a function of annealing time, the using solution volume was 0.1 mL. (**a**) 5 min; (**b**) 10 min; (**c**) 20 min; (**d**) 30 min.

The basic microstructure of the polycrystalline-CIS-chalcopyrite-based materials is complex, with a range of planar defects, such as micro-twins and stacking faults, facetted voids, pores, strong crystallographic texture, and variation in grain size. The surface morphologies of the CIS absorber layers under different annealing time are shown in Figures 5 and 6, which indicate that as the annealing time was changed and the surface morphologies were apparently changed as well. The grain sizes of the CIS absorber layers are dependent on the annealing time and usually many crystalline grains extend through the whole thin films’ thickness after annealing. As the results in Figures 5 and 6 were compared, the grain sizes of the 10 min- and 20 min-annealed CIS absorber layers were smaller than those of the 30 min-annealed ones. Even with smaller grain sizes, the optimal annealing time is 10 min and 20 min because the CIS absorber layers annealed at those time have the uniform grain size and are without abnormal grain growth. The CIS absorber layers annealed at 10 min and 20 min also have the densified morphology with less voids and pores. Figure 4 also shows that the 10 min-annealed CIS absorber layers have the largest TC value of (112) plane. For that, in this study, 10 min is the best annealing time for the CIS absorber layers.

However, the variations in the particle sizes of the CIS absorber layers are dependent on the KD1 concentration and grinding time and they are not easily calculated from the surface observation. In the past, the particle size can be estimated by using the Scherrer’s formula [13]:

$$D=\frac{k\lambda }{B\;\cos\theta }$$ (2)

where λ is the X-ray wavelength; *B* is the full width of height maximum of a diffraction peak; θ is the diffraction angle; and *k* is the Scherrer’s constant of the order unity for usual crystallization. The particle sizes of the 5-min-annealed samples were not calculated as they had abnormal grain sizes (samples with 0.05 mL IPA/CIS solution) or large voids (0.1 mL). A volume of 0.05 mL (0.1 mL) IPA/CIS composite was used, and annealing time was 5 min, 10 min, 20 min, and 30 min, the results are compared in Table 1. The average crystalline size of the nano particles was 36 (43) nm, 57 (71) nm, and 79 (95) nm, respectively. Evidently, the annealing time has a significant effect on the CIS absorber layer surface morphologies. As 0.05 mL IPA/CIS composite was used, and the annealing time was 5 min, the large grain sizes existed in a matrix of small particles, and the pores were distinctly observed; as the annealing time was increased to 10 min, only the small particles were revealed.

**Figure 7.**
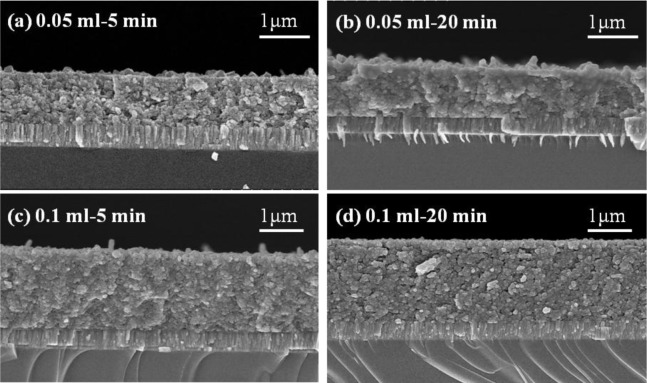
Cross section observations of the CIS absorber layers as a function of annealing time and using solution volume. (**a**) 0.05 mL–5 min; (**b**) 0.05 mL–20 min; (**c**) 0.1 mL–5 min; (**d**) 0.1 mL–20 min.

When the annealing time was more than 10 min, the particle sizes and densification apparently increased with increasing annealing time. As 0.1 mL IPA/CIS composite was used, and the annealing time was 5 min, larger grain sizes existed on the surface of the CIS absorber layers, and an un-densified structure and pores were also observed (Figure 7b); As the annealing time was changed from 10 min to 30 min, a more uniform grain size was obtained and the surface morphologies had no apparent change, but the densification increased with increasing annealing time. Those results suggest that 550 °C is high enough to improve the densification and grain growth of the CIS absorber layers, and the annealing time is an important factor to influence the crystallization and grain sizes of the CIS absorber layers. The detailed variation will be discussed later.

The thicknesses of the CIS absorber layers deposited with various volumes of the IPA/CIS composite, and annealed at 550 °C, at different times, are observed from the cross-section images of FESEM and the results are shown in Figure 7, and the details are compared in Table 1. As 0.05 mL IPA/CIS composite was used, the thicknesses of the CIS absorption layers were around 965 ± 30 nm; As 0.1 mL IPA/CIS composite was used, the thicknesses of the CIS absorption layers were around 1905 ± 50 nm. The results in Figure 7 reveal that the thickness of the CIS absorption layers with 0.1 mL IPA/CIS composite was increased to about double, as compared with the thickness of the samples with 0.05 mL IPA/CIS composite. Those results prove that if we control the used volume of the IPA/CIS composite, we can obtain the expected thickness of the CIS absorption layers by using the spray coating method. However, the small voids or pores were apparently observed in the 5-min-annealed 0.05 mL IPA/CIS samples, as compared with the other results shown in Figures 5 and 6.

Comparing the results in Figures 3 and 7, the effect of annealing time on the crystallinity, grain growth, and densification of the CIS absorption layers can be explained below. When annealing time was 5 min, as Figure 5a shows, the surface morphology of the CIS absorber layers using 0.05 mL IPA/CIS composite showed a bimodal non-uniform distribution of grain sizes, which exhibited exaggerated discontinuous grain in a fine-grained matrix. The abnormal grain growth (sometimes also called secondary re-crystallization) is characterized by the rapid growth of only a small number grains. This may result from the presence of small amount eutectic formed by the additive or composite itself [14]. The pores are also observed in Figure 5a and Figure 7a, these results are caused by that the abnormal grains grow at the expense of small ones, which result in the formation of new and larger voids where the small grains are originally located. As the annealing time of the 0.05 mL IPA/CIS composite is 10 min, the effect to cause the abnormal grain growths is not observed, as Figure 5b shows, and the CIS absorber layers show the densified structures. As no abnormal grain growth is formed in CIS absorber layers of the 0.05 ml IPA/CIS composite with the annealing time of 10 min, the diffraction intensity of (112) plane decreases. As annealing time is equal to and longer than 10 min, the grain sizes increase with increasing annealing time, for that the diffraction intensity of (112) plane also increases with increasing annealing time, as Figure 3a shows. Comparing the results in Figure 3b, Figure 6, Figure 7c,d, for 0.1 mL IPA/CIS composite, the abnormal grain growths are apparently observed on surface Figures 6a and 7d, and the abnormal grain growths are not observed as the annealing time is equal to and longer than 10 min.

**Figure 8.**
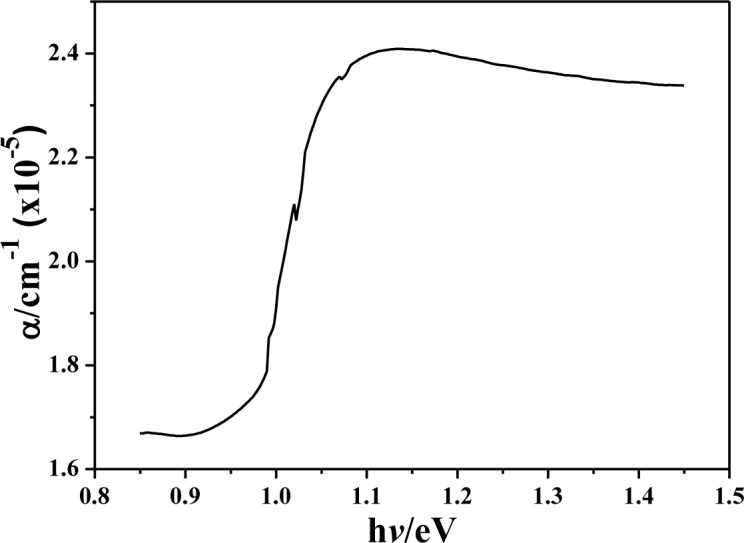
Photon energy dependences of the absorption coefficient for annealed CIS absorber layers.

Figure 8 shows the photon energy dependence of the absorption coefficient for the annealed CIS absorption layers. An absorption coefficient exceeding 10^5^ cm^−1^ is obtained by absorption spectroscopy measurements for all CIS absorber layers. As compared with other research, the order (10^5^ cm^−1^) of the absorption coefficient obtained in this study is same with that investigated by P. Luo *et al.* [15], and is higher than that investigated by N. Kavcar [16]. Those results suggest that the CIS thin films investigated in this study have a good optical quality. The plot (α*h*ν)^2^ *versus* (*h*ν) of the annealed CIS absorber layers is used to calculate the energy band gap of the annealed CIS absorber layers. It is well established that CIS thin films are a direct band gap semi-conductor with the band extrema located at the center of Brillouin [17]. The direct band gap of samples obtained from the plot of (α*h*ν)^2^ *versus* (*h*ν) is in the range of 1.028–1.045 eV, which is also compared in Table 1. The energy band gap had no apparent variations as the annealing temperature and IPA/CIS volume were changed. The small variation in the composition is the reason to cause this result.

**Figure 9.**
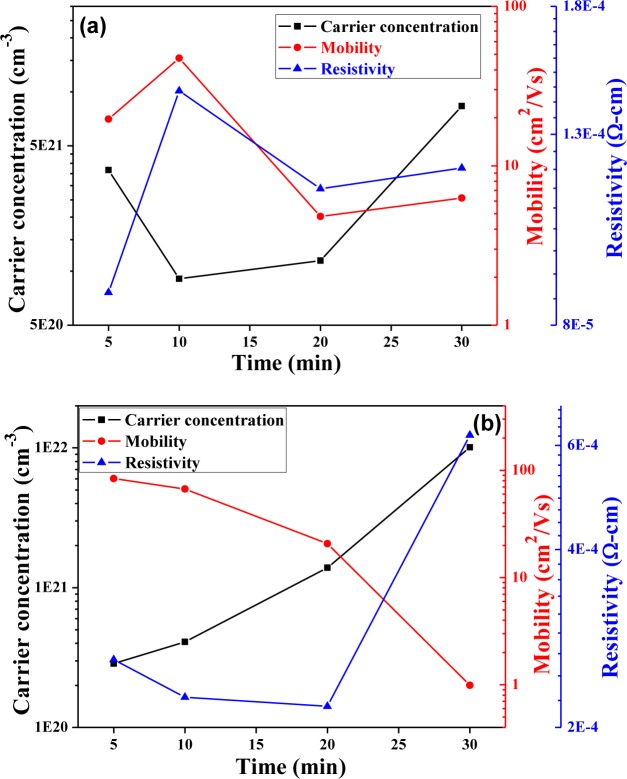
Carrier concentration (*n*), hall mobility (*μ*), and resistivity (*ρ*) of the CIS absorber layers as a function of annealing time, with using solution volume was (**a**) 0.05 mL; and (**b**) 0.1 mL, respectively.

Figure 9 shows, carrier mobility (*μ*), carrier concentration (*n*), and resistivity (*ρ*) of the CIS absorption layers as a function of annealing time. However, the Hall measurements prove the CIS absorber layers are with *p* type. As Figure 9a shows, as 0.05 mL IPA/CIS composite was used, the carrier concentration first decreased from 5-min-annealed samples and then slightly increased with a further increase in the annealing time. This result is matched the variations of crystallinity shown in Figure 3a and grain growth shown in Figure 5. However, as 0.1 mL IPA/CIS composite is used, the variation of carrier concentration is matched to that of crystallinity shown in Figure 3b and that of grain growth, shown in Figure 6. For that, the crystallinity and grain growth are the main reason to dominate the variations of carrier concentration. In addition, as 0.05 mL IPA/CIS composite was used the carrier concentration first decreased from 3.67 × 10^21^ cm^−3^ to 9.08 × 10^20^ cm^−3^ as annealing time was increased from 5 min to 10 min, and then linearly increased to 8.34 × 10^21^ cm^−3^ when the annealing time was 30 min, respectively. However, as 0.1 mL IPA/CIS composite was used, the carrier concentration linearly increased from 2.86 × 10^20^ cm^−3^ to 1.01 × 10^21^ cm^−3^ as annealing time was increased from 5 min to 30 min. The high crystallinity and larger grain growth are believed to be the reasons for causing the CIS absorber layers with high carrier concentration.

When the IPA/CIS composite is annealed to form the CIS absorber layers in a selenization furnace without extra compensation Se or H_2_Se, many defects result and inhibit electron movement. As the longer annealing time is used, the CIS absorber layers’ densification and crystallization can be enhanced. The increase in the numbers of defects and pores in the CIS absorber layer is the reason to cause the increase in the inhibiting of the barriers electron transportation [18], for that, the mobility apparently decreases, especially in the samples with 0.1 mL IPA/CIS composite.

Resistivity of the CIS absorption layers is proportional to the reciprocal of the product of carrier concentration n and mobility μ:

$$\begin{array}{c} \rho =1/\mathrm{ne\mu} \end{array}$$ (3)

As Equation (2) shows, both the carrier concentration and the carrier mobility contribute to the resistivity. As 0.05 mL IPA/CIS composite was used, the resistivity of CIS absorption layers were in the range of 8.69 × 10^−5^ Ω-cm–1.26 × 10^−4^ Ω-cm and the minimum value appeared in 5-min-annealed samples; As 0.1 mL IPA/CIS composite was used, the resistivity of CIS absorption layers using were in the range of 2.1× 10^−4^ Ω-cm–6.24 × 10^−4^ Ω-cm and the minimum value appeared in 20-min-annealed samples. For 0.05 mL IPA/CIS composite, the minimum resistivity of the CIS absorption layers is mainly caused by both the carrier concentration and the carrier mobility being at its maximum.

## 4. Conclusions

In this study, all the CIS absorber layers had the polycrystalline structure and were indicated in crystallographic planes in the chalcopyrite structure. As 0.05 mL IPA/CIS composite was used, the maximum TC value of (112) plane was revealed at 10-min-annealed CIS absorber layers; as 0.1 mL IPA/CIS composite was used, the maximum TC value of (112) plane of the CIS absorber layers had no apparent trend as annealing time was increased. The thicknesses of the CIS absorption layers for 0.05 mL IPA/CIS composite and 0.1 mL IPA/CIS composite were around 965 ± 30 nm and 1905 ± 53 nm, respectively. The 0.05 mL IPA/CIS composite had a minimum resistivity of 8.69 × 10^−5^ Ω-cm for the 5-min-annealed CIS absorption layers and 0.1 mL IPA/CIS composite had a minimum resistivity of 2.17 × 10^−4^ Ω-cm for 5 min-annealed CIS absorption layers. However, we proved that a non-vacuum SPM method and annealing process could be used to deposit the highly densified CIS absorber layers on Mo/glass substrates, and the CIS absorber layer characteristics were comparable to the sputter-deposited and co-evaporation ones.

## Figures and Tables

**Table 1.** Average particle sizes, average thickness, chemical composition, absorption coefficient, and energy band gap of the CuInSe_2_ (CIS) absorber layers as the functions of annealing time and used volume of CIS isopropyl alcohol (IPA/CIS) solution.

Time (min)-IPA/CIS volume (mL)	Average particle size (nm)	Average thickness (nm)	Chemical composition (Cu:In:Se)	Energy band gap (eV)
5-0.05	X	995	24.9:27.2:47.9	1.028
5-0.1	X	1852	25.0:27.1:47.9	1.031
10-0.05	36	935	25.3:27.3:47.4	1.036
10-0.1	43	1955	25.2:27.3:47.5	1.039
20-0.05	57	970	25.3:27.5:47.2	1.039
20-0.1	71	1868	25.4:27.5:47.1	1.041
30-0.05	79	978	25.5:27.7:46.8	1.042
30-0.1	95	1943	25.4:27.7:46.9	1.045

## References

[1] Powalla M., Voorwinden G., Hariskos D., Jackson P., Kniese R. (2009). Highly efficient CIS solar cells and modules made by the co-evaporation process. Thin Solid Films.

[2] Hsu C.Y., Huang P.C., Chen Y.Y., Wen D.C. (2013). Fabrication of a Cu(InGa)Se_2_ thin film photovoltaic absorber by rapid thermal annealing of CuGa/In precursors coated with a Se layer. Int. J. Photoenergy.

[3] Lin Y.C., Ke J.H., Yen W.T., Liang S.C., Wu C.H., Chiang C.T. (2011). Preparation and characterization of Cu(In,Ga)(Se,S)_2_ films without selenization by co-sputtering from Cu(In,Ga)Se_2_ quaternary and In_2_S_2_ targets. Appl. Surf. Sci.

[4] Probst V., Stetter W., Riedl W., Vogt H., Wendl M., Calwer H., Zweigart S., Ufert K.D., Freienstein B., Cerva H., Karg F.H. (2001). Rapid CIS-process for high efficiency PV-modules: development towards large area processing. Thin Solid Films.

[5] Song H.K., Jeong J.K., Kim H.J., Kim S.K., Yoon K.H. (2003). Fabrication of CuIn_1−_*_x_* Ga*_x_* Se_2_ thin film solar cells by sputtering and selenization process. Thin Solid Films.

[6] Park H., Kim S.C., Lee S.H., Koo J., Lee S.H., Jeon C.W., Yoon S., Kim W.K. (2011). Effect of precursor structure on Cu(InGa)Se_2_ formation by reactive annealing. Thin Solid Films.

[7] Shi J.H., Li Z.Q., Zhang D.W., Liu Q.Q., Sun Z., Huang S.M. (2011). Fabrication of Cu(In, Ga)Se_2_ thin films by sputtering from a single quaternary chalcogenide target. Prog. Photovolt.

[8] Guha P., Kundu S.N., Chaudhuri S., Pal A.K. (2002). Electron transport processes in CuIn_1−x_Ga_x_Se_2_ films prepared by four source co-evaporation technique. Mater. Chem. Phy.

[9] Saji V.S., Choi I.H., Lee C.W. (2011). Progress in electrodeposited absorber layer for CuIn_(1-x)_GaxSe_2_ (CIGS) solar cells. Sol. Energy.

[10] Eberspacher C., Fredric C., Pauls K., Serra J. (2001). Thin-film CIS alloy PV materials fabricated using non-vacuum, particles-based techniques. Thin Solid Films.

[11] Wu C.C., Yang C.F. (2013). Investigate the properties of nanostructured Li-doped NiO films using the modified spray pyrolysis method. Nanoscale Res. Lett.

[12] Huang H.H., Diao C.C., Yang C.F., Huang C.J. (2010). Effects of substrate temperatures on the crystallizations and microstructures of electron beam evaporation YSZ thin films. J. Alloys Compd.

[13] Cullity B.D., Stock S.R. (2001). Elements of X-Ray Diffraction.

[14] Yang C.F., Wu L., Wu T.S. (1992). A new sintering agent for BaTiO3: The binary BaO-CuO systems. J. Mater. Sci. Lett.

[15] Luo P., Yu P., Zuo R., Jin J., Ding Y., Song J., Chen Y. (2010). The preparation of CuInSe_2_ films by solvothermal route and non-vacuum spin-coating process. Phys. B.

[16] Kavcar N. (1998). Study of the sub-bandgap absorption and the optical transitions in CuInSe_2_ polycrystalline thin films. Sol. Energy Mater. Sol. Cells.

[17] Rabeh M.B., Chaglabou N., Kanzari M. (2009). Effect of antimony incorporation in CuInS_2_ thin films. Chalcogenide Lett.

[18] Igasaki Y., Saito H. (1991). Substrate temperature dependence of electrical properties of ZnO:Al epitaxial films on sapphire (1210). J. Appl. Phys.

